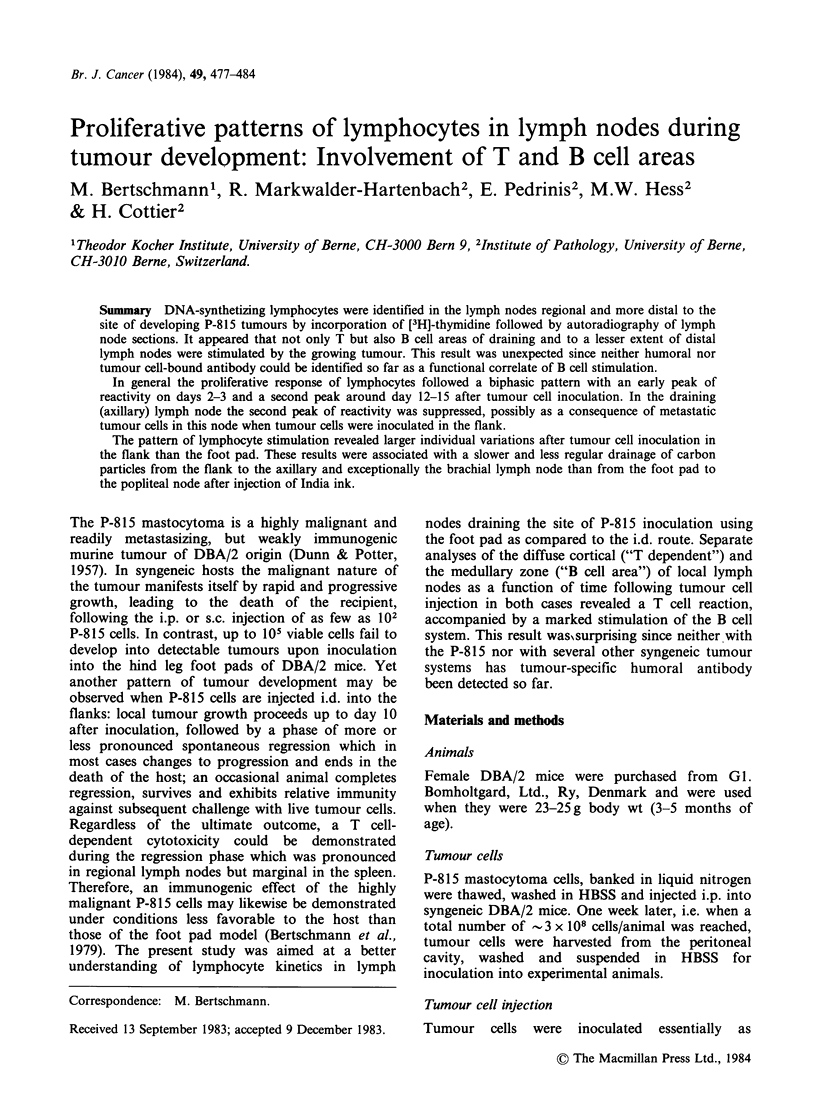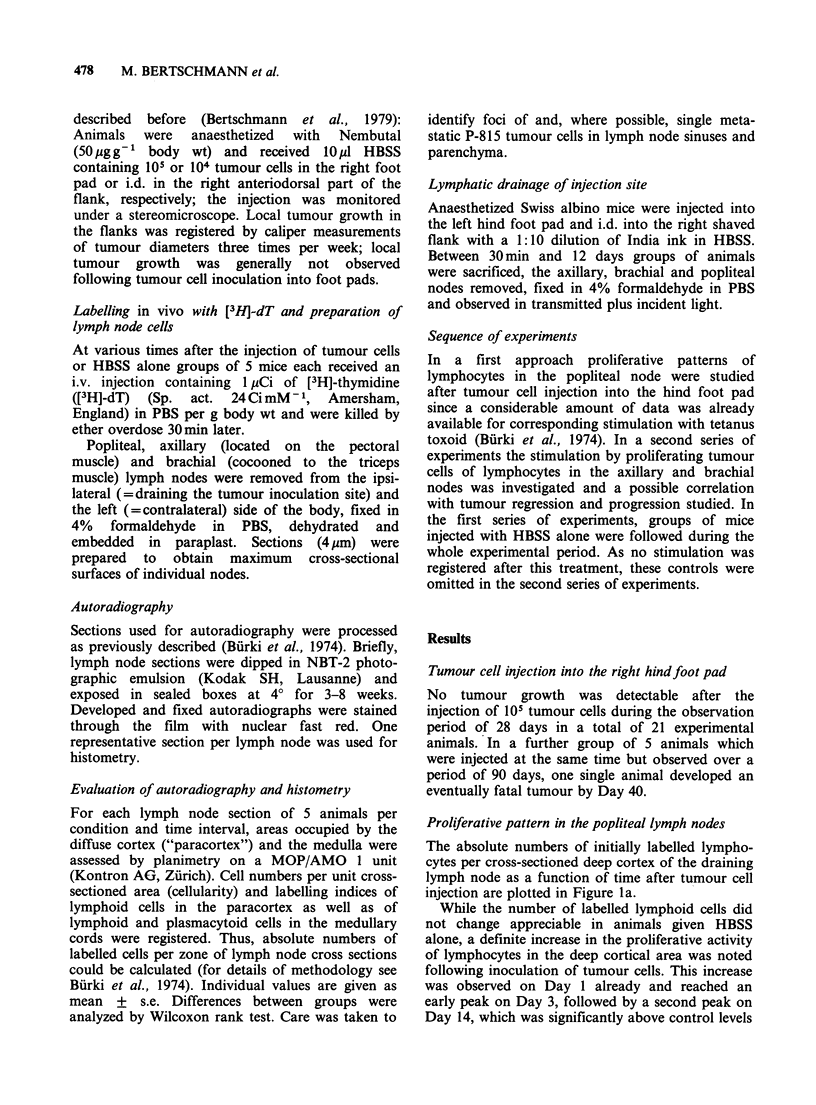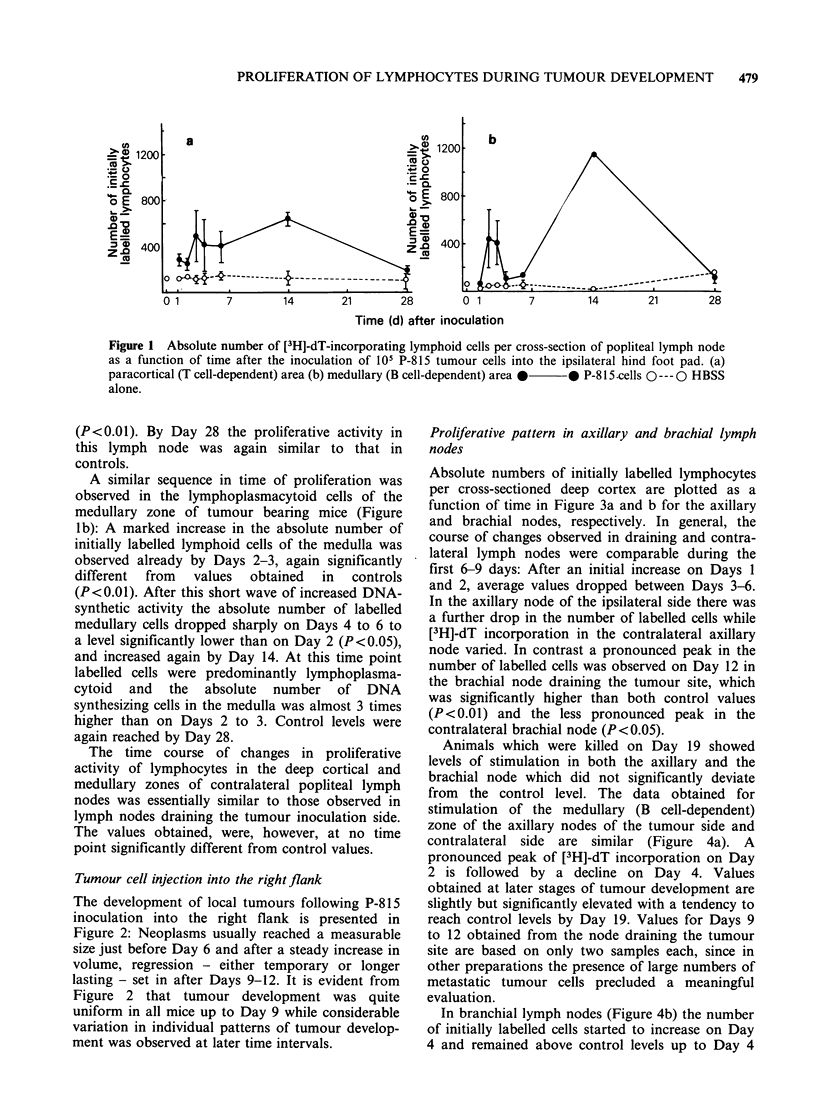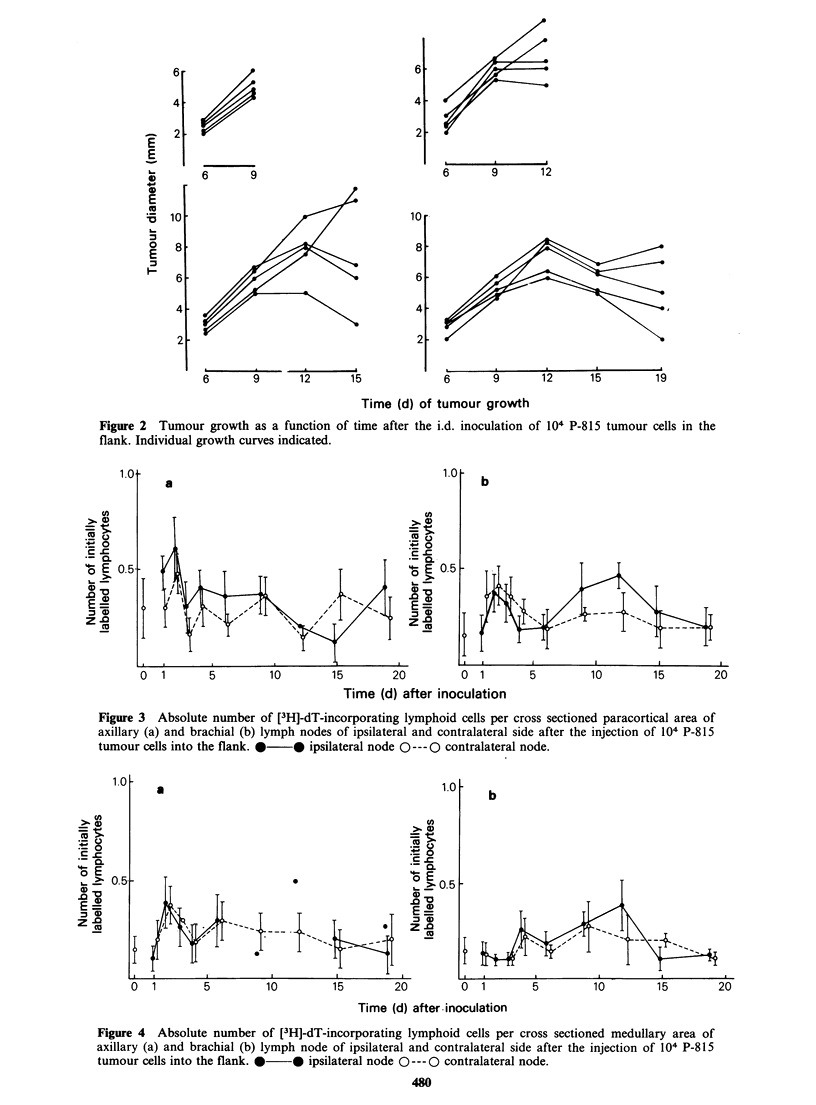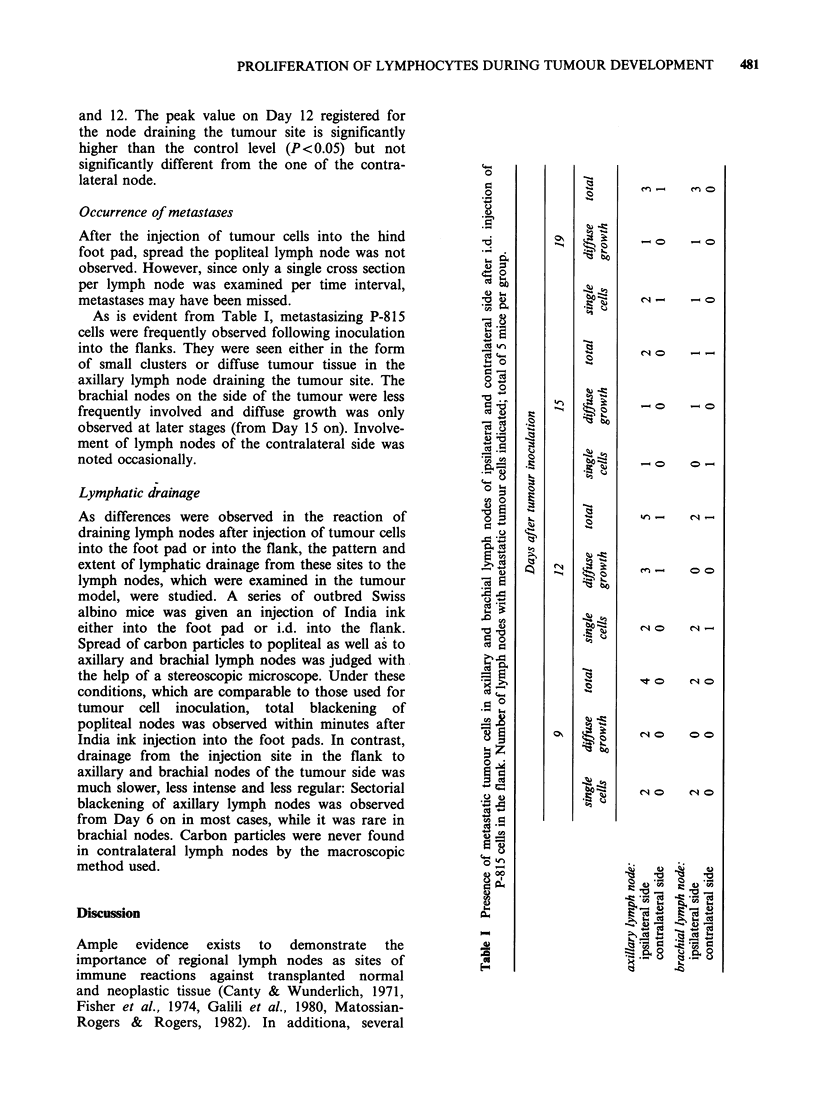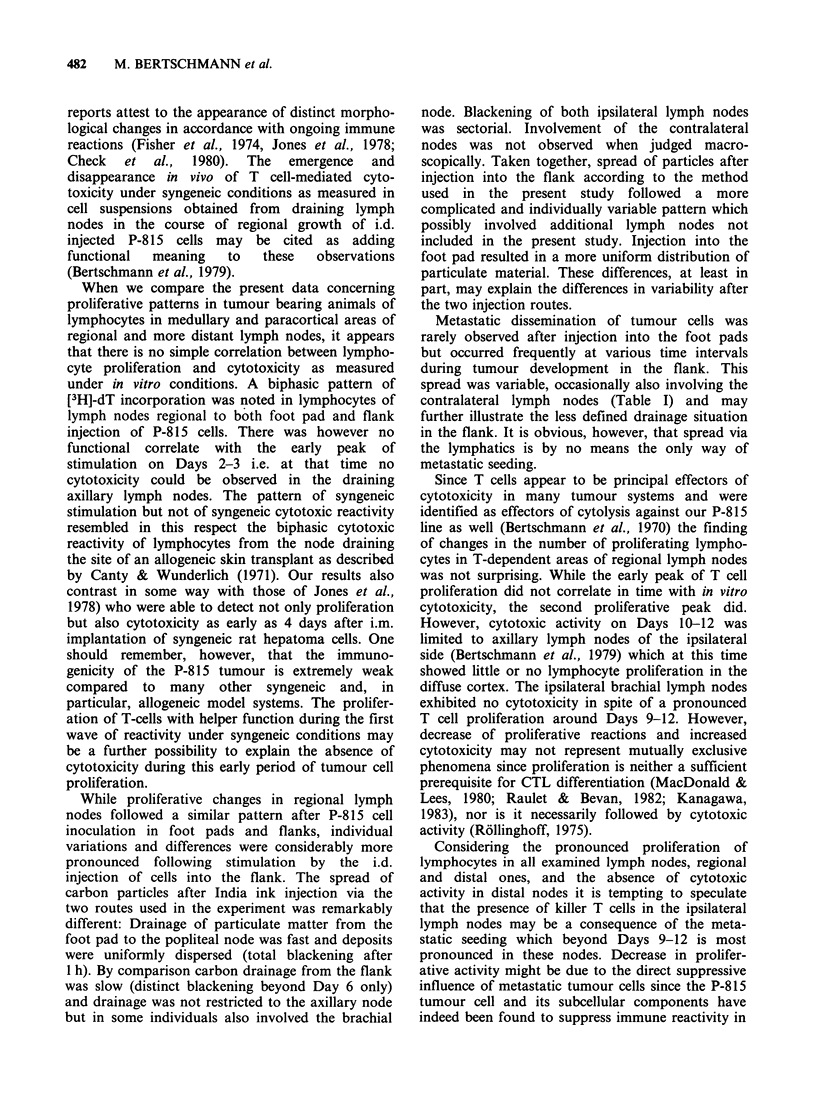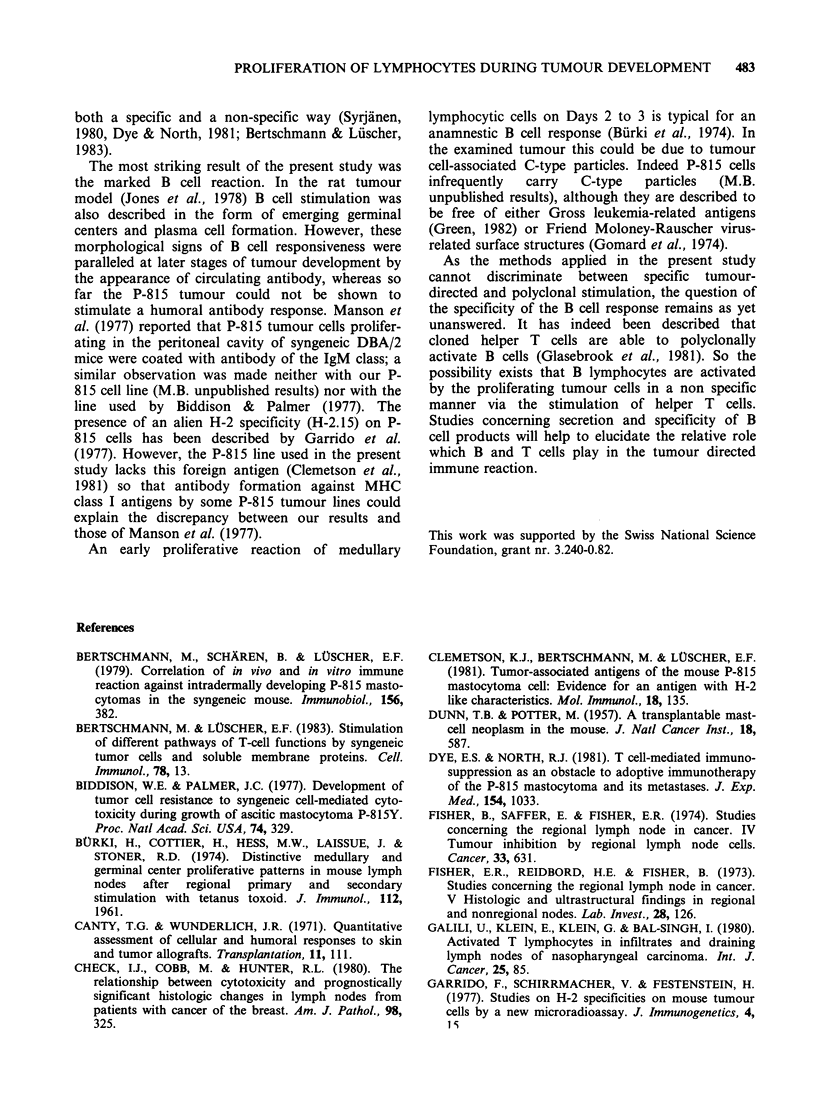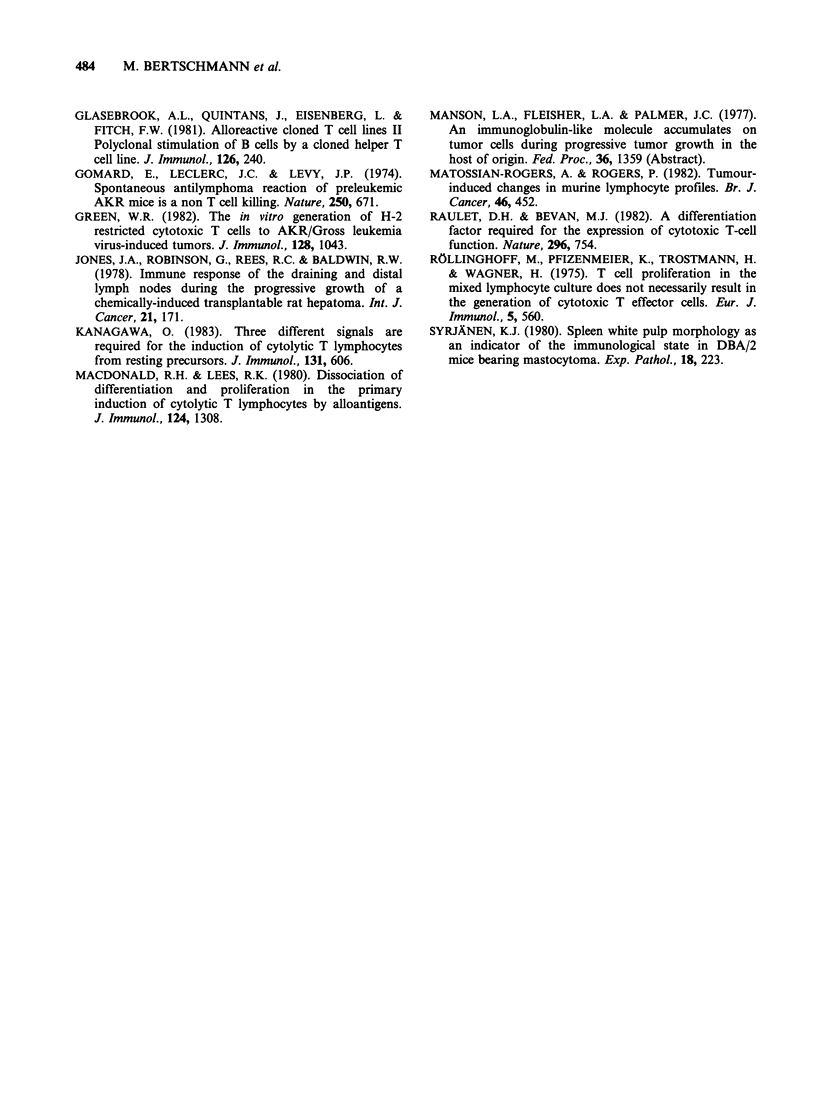# Proliferative patterns of lymphocytes in lymph nodes during tumour development: involvement of T and B cell areas.

**DOI:** 10.1038/bjc.1984.75

**Published:** 1984-04

**Authors:** M. Bertschmann, R. Markwalder-Hartenbach, E. Pedrinis, M. W. Hess, H. Cottier

## Abstract

DNA-synthetizing lymphocytes were identified in the lymph nodes regional and more distal to the site of developing P-815 tumours by incorporation of [3H]-thymidine followed by autoradiography of lymph node sections. It appeared that not only T but also B cell areas of draining and to a lesser extent of distal lymph nodes were stimulated by the growing tumour. This result was unexpected since neither humoral nor tumour cell-bound antibody could be identified so far as a functional correlate of B cell stimulation. In general the proliferative response of lymphocytes followed a biphasic pattern with an early peak of reactivity on days 2-3 and a second peak around day 12-15 after tumour cell inoculation. In the draining (axillary) lymph node the second peak of reactivity was suppressed, possibly as a consequence of metastatic tumour cells in this node when tumour cells were inoculated in the flank. The pattern of lymphocyte stimulation revealed larger individual variations after tumour cell inoculation in the flank than the foot pad. These results were associated with a slower and less regular drainage of carbon particles from the flank to the axillary and exceptionally the brachial lymph node than from the foot pad to the popliteal node after injection of India ink.


*Br. J. Cancer* (1984), **49**, 477–484

### Proliferative patterns of lymphocytes in lymph nodes during tumour development: Involvement of T and B cell areas

M. Bertschmann[1], R. Markwalder-Hartenbach[2], E. Pedrinis[2], M.W. Hess[2] & H. Cottier[2]

[1]*Theodor Kocher Institute, University of Berne, CH-3000 Bern 9,* [2]*Institute of Pathology, University of Berne, CH-3010 Berne, Switzerland.*

**Summary** DNA-synthetizing lymphocytes were identified in the lymph nodes regional and more distal to the site of developing P-815 tumours by incorporation of [$^3$H]-thymidine followed by autoradiography of lymph node sections. It appeared that not only T but also B cell areas of draining and to a lesser extent of distal lymph nodes were stimulated by the growing tumour. This result was unexpected since neither humoral nor tumour cell-bound antibody could be identified so far as a functional correlate of B cell stimulation.

In general the proliferative response of lymphocytes followed a biphasic pattern with an early peak of reactivity on days 2–3 and a second peak around day 12–15 after tumour cell inoculation. In the draining (axillary) lymph node the second peak of reactivity was suppressed, possibly as a consequence of metastatic tumour cells in this node when tumour cells were inoculated in the flank.

The pattern of lymphocyte stimulation revealed larger individual variations after tumour cell inoculation in the flank than the foot pad. These results were associated with a slower and less regular drainage of carbon particles from the flank to the axillary and exceptionally the brachial lymph node than from the foot pad to the popliteal node after injection of India ink.

The P-815 mastocytoma is a highly malignant and readily metastasizing, but weakly immunogenic murine tumour of DBA/2 origin (Dunn & Potter, 1957). In syngeneic hosts the malignant nature of the tumour manifests itself by rapid and progressive growth, leading to the death of the recipient, following the i.p. or s.c. injection of as few as $10^2$ P-815 cells. In contrast, up to $10^5$ viable cells fail to develop into detectable tumours upon inoculation into the hind leg foot pads of DBA/2 mice. Yet another pattern of tumour development may be observed when P-815 cells are injected i.d. into the flanks: local tumour growth proceeds up to day 10 after inoculation, followed by a phase of more or less pronounced spontaneous regression which in most cases changes to progression and ends in the death of the host; an occasional animal completes regression, survives and exhibits relative immunity against subsequent challenge with live tumour cells. Regardless of the ultimate outcome, a T cell-dependent cytotoxicity could be demonstrated during the regression phase which was pronounced in regional lymph nodes but marginal in the spleen. Therefore, an immunogenic effect of the highly malignant P-815 cells may likewise be demonstrated under conditions less favorable to the host than those of the foot pad model (Bertschmann *et al.*, 1979). The present study was aimed at a better understanding of lymphocyte kinetics in lymph nodes draining the site of P-815 inoculation using the foot pad as compared to the i.d. route. Separate analyses of the diffuse cortical ("T dependent") and the medullary zone ("B cell area") of local lymph nodes as a function of time following tumour cell injection in both cases revealed a T cell reaction, accompanied by a marked stimulation of the B cell system. This result was surprising since neither with the P-815 nor with several other syngeneic tumour systems has tumour-specific humoral antibody been detected so far.

Correspondence: M. Bertschmann.

Received 13 September 1983; accepted 9 December 1983.

### Materials and methods

### *Animals*

Female DBA/2 mice were purchased from G1. Bomholtgard, Ltd., Ry, Denmark and were used when they were 23–25 g body wt (3–5 months of age).

### *Tumour cells*

P-815 mastocytoma cells, banked in liquid nitrogen were thawed, washed in HBSS and injected i.p. into syngeneic DBA/2 mice. One week later, i.e. when a total number of $\sim 3 \times 10^8$ cells/animal was reached, tumour cells were harvested from the peritoneal cavity, washed and suspended in HBSS for inoculation into experimental animals.

### *Tumour cell injection*

Tumour cells were inoculated essentially as

© The Macmillan Press Ltd., 1984

described before (Bertschmann *et al.*, 1979): Animals were anaesthetized with Nembutal ($50\,\mu g\,g^{-1}$ body wt) and received $10\,\mu l$ HBSS containing $10^5$ or $10^4$ tumour cells in the right foot pad or i.d. in the right anteriodorsal part of the flank, respectively; the injection was monitored under a stereomicroscope. Local tumour growth in the flanks was registered by caliper measurements of tumour diameters three times per week; local tumour growth was generally not observed following tumour cell inoculation into foot pads.

*Labelling* in vivo *with [$^3H$]-dT and preparation of lymph node cells*

At various times after the injection of tumour cells or HBSS alone groups of 5 mice each received an i.v. injection containing $1\,\mu Ci$ of [$^3$H]-thymidine ([$^3$H]-dT) (Sp. act. $24\,Ci\,mM^{-1}$, Amersham, England) in PBS per g body wt and were killed by ether overdose 30 min later.

Popliteal, axillary (located on the pectoral muscle) and brachial (cocooned to the triceps muscle) lymph nodes were removed from the ipsilateral (=draining the tumour inoculation site) and the left (=contralateral) side of the body, fixed in 4% formaldehyde in PBS, dehydrated and embedded in paraplast. Sections ($4\,\mu m$) were prepared to obtain maximum cross-sectional surfaces of individual nodes.

*Autoradiography*

Sections used for autoradiography were processed as previously described (Bürki *et al.*, 1974). Briefly, lymph node sections were dipped in NBT-2 photographic emulsion (Kodak SH, Lausanne) and exposed in sealed boxes at 4° for 3–8 weeks. Developed and fixed autoradiographs were stained through the film with nuclear fast red. One representative section per lymph node was used for histometry.

*Evaluation of autoradiography and histometry*

For each lymph node section of 5 animals per condition and time interval, areas occupied by the diffuse cortex ("paracortex") and the medulla were assessed by planimetry on a MOP/AMO 1 unit (Kontron AG, Zürich). Cell numbers per unit cross-sectioned area (cellularity) and labelling indices of lymphoid cells in the paracortex as well as of lymphoid and plasmacytoid cells in the medullary cords were registered. Thus, absolute numbers of labelled cells per zone of lymph node cross sections could be calculated (for details of methodology see Bürki *et al.*, 1974). Individual values are given as mean ± s.e. Differences between groups were analyzed by Wilcoxon rank test. Care was taken to identify foci of and, where possible, single metastatic P-815 tumour cells in lymph node sinuses and parenchyma.

*Lymphatic drainage of injection site*

Anaesthetized Swiss albino mice were injected into the left hind foot pad and i.d. into the right shaved flank with a 1:10 dilution of India ink in HBSS. Between 30 min and 12 days groups of animals were sacrificed, the axillary, brachial and popliteal nodes removed, fixed in 4% formaldehyde in PBS and observed in transmitted plus incident light.

*Sequence of experiments*

In a first approach proliferative patterns of lymphocytes in the popliteal node were studied after tumour cell injection into the hind foot pad since a considerable amount of data was already available for corresponding stimulation with tetanus toxoid (Bürki *et al.*, 1974). In a second series of experiments the stimulation by proliferating tumour cells of lymphocytes in the axillary and brachial nodes was investigated and a possible correlation with tumour regression and progression studied. In the first series of experiments, groups of mice injected with HBSS alone were followed during the whole experimental period. As no stimulation was registered after this treatment, these controls were omitted in the second series of experiments.

### Results

*Tumour cell injection into the right hind foot pad*

No tumour growth was detectable after the injection of $10^5$ tumour cells during the observation period of 28 days in a total of 21 experimental animals. In a further group of 5 animals which were injected at the same time but observed over a period of 90 days, one single animal developed an eventually fatal tumour by Day 40.

*Proliferative pattern in the popliteal lymph nodes*

The absolute numbers of initially labelled lymphocytes per cross-sectioned deep cortex of the draining lymph node as a function of time after tumour cell injection are plotted in Figure 1a.

While the number of labelled lymphoid cells did not change appreciable in animals given HBSS alone, a definite increase in the proliferative activity of lymphocytes in the deep cortical area was noted following inoculation of tumour cells. This increase was observed on Day 1 already and reached an early peak on Day 3, followed by a second peak on Day 14, which was significantly above control levels

**Figure 1** Absolute number of [$^{3}$H]-dT-incorporating lymphoid cells per cross-section of popliteal lymph node as a function of time after the inoculation of $10^5$ P-815 tumour cells into the ipsilateral hind foot pad. (a) paracortical (T cell-dependent) area (b) medullary (B cell-dependent) area ●———● P-815-cells ○---○ HBSS alone.

($P<0.01$). By Day 28 the proliferative activity in this lymph node was again similar to that in controls.

A similar sequence in time of proliferation was observed in the lymphoplasmacytoid cells of the medullary zone of tumour bearing mice (Figure 1b): A marked increase in the absolute number of initially labelled lymphoid cells of the medulla was observed already by Days 2–3, again significantly different from values obtained in controls ($P<0.01$). After this short wave of increased DNA-synthetic activity the absolute number of labelled medullary cells dropped sharply on Days 4 to 6 to a level significantly lower than on Day 2 ($P<0.05$), and increased again by Day 14. At this time point labelled cells were predominantly lymphoplasmacytoid and the absolute number of DNA synthesizing cells in the medulla was almost 3 times higher than on Days 2 to 3. Control levels were again reached by Day 28.

The time course of changes in proliferative activity of lymphocytes in the deep cortical and medullary zones of contralateral popliteal lymph nodes was essentially similar to those observed in lymph nodes draining the tumour inoculation side. The values obtained, were, however, at no time point significantly different from control values.

*Tumour cell injection into the right flank*

The development of local tumours following P-815 inoculation into the right flank is presented in Figure 2: Neoplasms usually reached a measurable size just before Day 6 and after a steady increase in volume, regression – either temporary or longer lasting – set in after Days 9–12. It is evident from Figure 2 that tumour development was quite uniform in all mice up to Day 9 while considerable variation in individual patterns of tumour development was observed at later time intervals.

*Proliferative pattern in axillary and brachial lymph nodes*

Absolute numbers of initially labelled lymphocytes per cross-sectioned deep cortex are plotted as a function of time in Figure 3a and b for the axillary and brachial nodes, respectively. In general, the course of changes observed in draining and contralateral lymph nodes were comparable during the first 6–9 days: After an initial increase on Days 1 and 2, average values dropped between Days 3–6. In the axillary node of the ipsilateral side there was a further drop in the number of labelled cells while [$^{3}$H]-dT incorporation in the contralateral axillary node varied. In contrast a pronounced peak in the number of labelled cells was observed on Day 12 in the brachial node draining the tumour site, which was significantly higher than both control values ($P<0.01$) and the less pronounced peak in the contralateral brachial node ($P<0.05$).

Animals which were killed on Day 19 showed levels of stimulation in both the axillary and the brachial node which did not significantly deviate from the control level. The data obtained for stimulation of the medullary (B cell-dependent) zone of the axillary nodes of the tumour side and contralateral side are similar (Figure 4a). A pronounced peak of [$^{3}$H]-dT incorporation on Day 2 is followed by a decline on Day 4. Values obtained at later stages of tumour development are slightly but significantly elevated with a tendency to reach control levels by Day 19. Values for Days 9 to 12 obtained from the node draining the tumour site are based on only two samples each, since in other preparations the presence of large numbers of metastatic tumour cells precluded a meaningful evaluation.

In branchial lymph nodes (Figure 4b) the number of initially labelled cells started to increase on Day 4 and remained above control levels up to Day 4

**Figure 2** Tumour growth as a function of time after the i.d. inoculation of $10^4$ P-815 tumour cells in the flank. Individual growth curves indicated.

**Figure 3** Absolute number of [$^3$H]-dT-incorporating lymphoid cells per cross sectioned paracortical area of axillary (a) and brachial (b) lymph nodes of ipsilateral and contralateral side after the injection of $10^4$ P-815 tumour cells into the flank. ●——● ipsilateral node ○---○ contralateral node.

**Figure 4** Absolute number of [$^3$H]-dT-incorporating lymphoid cells per cross sectioned medullary area of axillary (a) and brachial (b) lymph node of ipsilateral and contralateral side after the injection of $10^4$ P-815 tumour cells into the flank. ●——● ipsilateral node ○---○ contralateral node.

and 12. The peak value on Day 12 registered for the node draining the tumour site is significantly higher than the control level ($P<0.05$) but not significantly different from the one of the contralateral node.

*Occurrence of metastases*

After the injection of tumour cells into the hind foot pad, spread the popliteal lymph node was not observed. However, since only a single cross section per lymph node was examined per time interval, metastases may have been missed.

As is evident from Table I, metastasizing P-815 cells were frequently observed following inoculation into the flanks. They were seen either in the form of small clusters or diffuse tumour tissue in the axillary lymph node draining the tumour site. The brachial nodes on the side of the tumour were less frequently involved and diffuse growth was only observed at later stages (from Day 15 on). Involvement of lymph nodes of the contralateral side was noted occasionally.

*Lymphatic drainage*

As differences were observed in the reaction of draining lymph nodes after injection of tumour cells into the foot pad or into the flank, the pattern and extent of lymphatic drainage from these sites to the lymph nodes, which were examined in the tumour model, were studied. A series of outbred Swiss albino mice was given an injection of India ink either into the foot pad or i.d. into the flank. Spread of carbon particles to popliteal as well as to axillary and brachial lymph nodes was judged with the help of a stereoscopic microscope. Under these conditions, which are comparable to those used for tumour cell inoculation, total blackening of popliteal nodes was observed within minutes after India ink injection into the foot pads. In contrast, drainage from the injection site in the flank to axillary and brachial nodes of the tumour side was much slower, less intense and less regular: Sectorial blackening of axillary lymph nodes was observed from Day 6 on in most cases, while it was rare in brachial nodes. Carbon particles were never found in contralateral lymph nodes by the macroscopic method used.

**Table I** Presence of metastatic tumour cells in axillary and brachial lymph nodes of ipsilateral and contralateral side after i.d. injection of P-815 cells in the flank. Number of lymph nodes with metastatic tumour cells indicated; total of 5 mice per group.

| | *Days after tumour inoculation* | | | | | | | | | | | |
|---|---|---|---|---|---|---|---|---|---|---|---|---|
| | *9* | | | *12* | | | *15* | | | *19* | | |
| | *single cells* | *diffuse growth* | *total* | *single cells* | *diffuse growth* | *total* | *single cells* | *diffuse growth* | *total* | *single cells* | *diffuse growth* | *total* |
| *axillary lymph node:* | | | | | | | | | | | | |
| ipsilateral side | 2 | 2 | 4 | 2 | 3 | 5 | 1 | 1 | 2 | 2 | 1 | 3 |
| contralateral side | 0 | 0 | 0 | 0 | 1 | 1 | 0 | 0 | 0 | 1 | 0 | 1 |
| *brachial lymph node:* | | | | | | | | | | | | |
| ipsilateral side | 2 | 0 | 2 | 2 | 0 | 2 | 0 | 1 | 1 | 1 | 1 | 3 |
| contralateral side | 0 | 0 | 0 | 1 | 0 | 1 | 1 | 0 | 1 | 0 | 0 | 0 |

### Discussion

Ample evidence exists to demonstrate the importance of regional lymph nodes as sites of immune reactions against transplanted normal and neoplastic tissue (Canty & Wunderlich, 1971, Fisher *et al.*, 1974, Galili *et al.*, 1980, Matossian-Rogers & Rogers, 1982). In additiona, several reports attest to the appearance of distinct morphological changes in accordance with ongoing immune reactions (Fisher *et al.*, 1974, Jones *et al.*, 1978; Check *et al.*, 1980). The emergence and disappearance *in vivo* of T cell-mediated cytotoxicity under syngeneic conditions as measured in cell suspensions obtained from draining lymph nodes in the course of regional growth of i.d. injected P-815 cells may be cited as adding functional meaning to these observations (Bertschmann *et al.*, 1979).

When we compare the present data concerning proliferative patterns in tumour bearing animals of lymphocytes in medullary and paracortical areas of regional and more distant lymph nodes, it appears that there is no simple correlation between lymphocyte proliferation and cytotoxicity as measured under *in vitro* conditions. A biphasic pattern of [$^3$H]-dT incorporation was noted in lymphocytes of lymph nodes regional to both foot pad and flank injection of P-815 cells. There was however no functional correlate with the early peak of stimulation on Days 2–3 i.e. at that time no cytotoxicity could be observed in the draining axillary lymph nodes. The pattern of syngeneic stimulation but not of syngeneic cytotoxic reactivity resembled in this respect the biphasic cytotoxic reactivity of lymphocytes from the node draining the site of an allogeneic skin transplant as described by Canty & Wunderlich (1971). Our results also contrast in some way with those of Jones *et al.*, 1978) who were able to detect not only proliferation but also cytotoxicity as early as 4 days after i.m. implantation of syngeneic rat hepatoma cells. One should remember, however, that the immunogenicity of the P-815 tumour is extremely weak compared to many other syngeneic and, in particular, allogeneic model systems. The proliferation of T-cells with helper function during the first wave of reactivity under syngeneic conditions may be a further possibility to explain the absence of cytotoxicity during this early period of tumour cell proliferation.

While proliferative changes in regional lymph nodes followed a similar pattern after P-815 cell inoculation in foot pads and flanks, individual variations and differences were considerably more pronounced following stimulation by the i.d. injection of cells into the flank. The spread of carbon particles after India ink injection via the two routes used in the experiment was remarkably different: Drainage of particulate matter from the foot pad to the popliteal node was fast and deposits were uniformly dispersed (total blackening after 1 h). By comparison carbon drainage from the flank was slow (distinct blackening beyond Day 6 only) and drainage was not restricted to the axillary node but in some individuals also involved the brachial node. Blackening of both ipsilateral lymph nodes was sectorial. Involvement of the contralateral nodes was not observed when judged macroscopically. Taken together, spread of particles after injection into the flank according to the method used in the present study followed a more complicated and individually variable pattern which possibly involved additional lymph nodes not included in the present study. Injection into the foot pad resulted in a more uniform distribution of particulate material. These differences, at least in part, may explain the differences in variability after the two injection routes.

Metastatic dissemination of tumour cells was rarely observed after injection into the foot pads but occurred frequently at various time intervals during tumour development in the flank. This spread was variable, occasionally also involving the contralateral lymph nodes (Table I) and may further illustrate the less defined drainage situation in the flank. It is obvious, however, that spread via the lymphatics is by no means the only way of metastatic seeding.

Since T cells appear to be principal effectors of cytotoxicity in many tumour systems and were identified as effectors of cytolysis against our P-815 line as well (Bertschmann *et al.*, 1970) the finding of changes in the number of proliferating lymphocytes in T-dependent areas of regional lymph nodes was not surprising. While the early peak of T cell proliferation did not correlate in time with *in vitro* cytotoxicity, the second proliferative peak did. However, cytotoxic activity on Days 10–12 was limited to axillary lymph nodes of the ipsilateral side (Bertschmann *et al.*, 1979) which at this time showed little or no lymphocyte proliferation in the diffuse cortex. The ipsilateral brachial lymph nodes exhibited no cytotoxicity in spite of a pronounced T cell proliferation around Days 9–12. However, decrease of proliferative reactions and increased cytotoxicity may not represent mutually exclusive phenomena since proliferation is neither a sufficient prerequisite for CTL differentiation (MacDonald & Lees, 1980; Raulet & Bevan, 1982; Kanagawa, 1983), nor is it necessarily followed by cytotoxic activity (Röllinghoff, 1975).

Considering the pronounced proliferation of lymphocytes in all examined lymph nodes, regional and distal ones, and the absence of cytotoxic activity in distal nodes it is tempting to speculate that the presence of killer T cells in the ipsilateral lymph nodes may be a consequence of the metastatic seeding which beyond Days 9–12 is most pronounced in these nodes. Decrease in proliferative activity might be due to the direct suppressive influence of metastatic tumour cells since the P-815 tumour cell and its subcellular components have indeed been found to suppress immune reactivity in both a specific and a non-specific way (Syrjänen, 1980, Dye & North, 1981; Bertschmann & Lüscher, 1983).

The most striking result of the present study was the marked B cell reaction. In the rat tumour model (Jones *et al.*, 1978) B cell stimulation was also described in the form of emerging germinal centers and plasma cell formation. However, these morphological signs of B cell responsiveness were paralleled at later stages of tumour development by the appearance of circulating antibody, whereas so far the P-815 tumour could not be shown to stimulate a humoral antibody response. Manson *et al.* (1977) reported that P-815 tumour cells proliferating in the peritoneal cavity of syngeneic DBA/2 mice were coated with antibody of the IgM class; a similar observation was made neither with our P-815 cell line (M.B. unpublished results) nor with the line used by Biddison & Palmer (1977). The presence of an alien H-2 specificity (H-2.15) on P-815 cells has been described by Garrido *et al.* (1977). However, the P-815 line used in the present study lacks this foreign antigen (Clemetson *et al.*, 1981) so that antibody formation against MHC class I antigens by some P-815 tumour lines could explain the discrepancy between our results and those of Manson *et al.* (1977).

An early proliferative reaction of medullary lymphocytic cells on Days 2 to 3 is typical for an anamnestic B cell response (Bürki *et al.*, 1974). In the examined tumour this could be due to tumour cell-associated C-type particles. Indeed P-815 cells infrequently carry C-type particles (M.B. unpublished results), although they are described to be free of either Gross leukemia-related antigens (Green, 1982) or Friend Moloney-Rauscher virus-related surface structures (Gomard *et al.*, 1974).

As the methods applied in the present study cannot discriminate between specific tumour-directed and polyclonal stimulation, the question of the specificity of the B cell response remains as yet unanswered. It has indeed been described that cloned helper T cells are able to polyclonally activate B cells (Glasebrook *et al.*, 1981). So the possibility exists that B lymphocytes are activated by the proliferating tumour cells in a non specific manner via the stimulation of helper T cells. Studies concerning secretion and specificity of B cell products will help to elucidate the relative role which B and T cells play in the tumour directed immune reaction.

This work was supported by the Swiss National Science Foundation, grant nr. 3.240-0.82.

### References

BERTSCHMANN, M., SCHÄREN, B. & LÜSCHER, E.F. (1979). Correlation of *in vivo* and *in vitro* immune reaction against intradermally developing P-815 mastocytomas in the syngeneic mouse. *Immunobiol.*, **156**, 382.

BERTSCHMANN, M. & LÜSCHER, E.F. (1983). Stimulation of different pathways of T-cell functions by syngeneic tumor cells and soluble membrane proteins. *Cell. Immunol.*, **78**, 13.

BIDDISON, W.E. & PALMER, J.C. (1977). Development of tumor cell resistance to syngeneic cell-mediated cytotoxicity during growth of ascitic mastocytoma P-815Y. *Proc. Natl Acad. Sci. USA*, **74**, 329.

BÜRKI, H., COTTIER, H., HESS, M.W., LAISSUE, J. & STONER, R.D. (1974). Distinctive medullary and germinal center proliferative patterns in mouse lymph nodes after regional primary and secondary stimulation with tetanus toxoid. *J. Immunol.*, **112**, 1961.

CANTY, T.G. & WUNDERLICH, J.R. (1971). Quantitative assessment of cellular and humoral responses to skin and tumor allografts. *Transplantation*, **11**, 111.

CHECK, I.J., COBB, M. & HUNTER, R.L. (1980). The relationship between cytotoxicity and prognostically significant histologic changes in lymph nodes from patients with cancer of the breast. *Am. J. Pathol.*, **98**, 325.

CLEMETSON, K.J., BERTSCHMANN, M. & LÜSCHER, E.F. (1981). Tumor-associated antigens of the mouse P-815 mastocytoma cell: Evidence for an antigen with H-2 like characteristics. *Mol. Immunol.*, **18**, 135.

DUNN, T.B. & POTTER, M. (1957). A transplantable mast-cell neoplasm in the mouse. *J. Natl Cancer Inst.*, **18**, 587.

DYE, E.S. & NORTH, R.J. (1981). T cell-mediated immunosuppression as an obstacle to adoptive immunotherapy of the P-815 mastocytoma and its metastases. *J. Exp. Med.*, **154**, 1033.

FISHER, B., SAFFER, E. & FISHER, E.R. (1974). Studies concerning the regional lymph node in cancer. IV Tumour inhibition by regional lymph node cells. *Cancer*, **33**, 631.

FISHER, E.R., REIDBORD, H.E. & FISHER, B. (1973). Studies concerning the regional lymph node in cancer. V Histologic and ultrastructural findings in regional and nonregional nodes. *Lab. Invest.*, **28**, 126.

GALILI, U., KLEIN, E., KLEIN, G. & BAL-SINGH, I. (1980). Activated T lymphocytes in infiltrates and draining lymph nodes of nasopharyngeal carcinoma. *Int. J. Cancer*, **25**, 85.

GARRIDO, F., SCHIRRMACHER, V. & FESTENSTEIN, H. (1977). Studies on H-2 specificities on mouse tumour cells by a new microradioassay. *J. Immunogenetics*, **4**, 15.

GLASEBROOK, A.L., QUINTANS, J., EISENBERG, L. & FITCH, F.W. (1981). Alloreactive cloned T cell lines II Polyclonal stimulation of B cells by a cloned helper T cell line. *J. Immunol.*, **126**, 240.

GOMARD, E., LECLERC, J.C. & LEVY, J.P. (1974). Spontaneous antilymphoma reaction of preleukemic AKR mice is a non T cell killing. *Nature*, **250**, 671.

GREEN, W.R. (1982). The *in vitro* generation of H-2 restricted cytotoxic T cells to AKR/Gross leukemia virus-induced tumors. *J. Immunol.*, **128**, 1043.

JONES, J.A., ROBINSON, G., REES, R.C. & BALDWIN, R.W. (1978). Immune response of the draining and distal lymph nodes during the progressive growth of a chemically-induced transplantable rat hepatoma. *Int. J. Cancer*, **21**, 171.

KANAGAWA, O. (1983). Three different signals are required for the induction of cytolytic T lymphocytes from resting precursors. *J. Immunol.*, **131**, 606.

MACDONALD, R.H. & LEES, R.K. (1980). Dissociation of differentiation and proliferation in the primary induction of cytolytic T lymphocytes by alloantigens. *J. Immunol.*, **124**, 1308.

MANSON, L.A., FLEISHER, L.A. & PALMER, J.C. (1977). An immunoglobulin-like molecule accumulates on tumor cells during progressive tumor growth in the host of origin. *Fed. Proc.*, **36**, 1359 (Abstract).

MATOSSIAN-ROGERS, A. & ROGERS, P. (1982). Tumour-induced changes in murine lymphocyte profiles. *Br. J. Cancer*, **46**, 452.

RAULET, D.H. & BEVAN, M.J. (1982). A differentiation factor required for the expression of cytotoxic T-cell function. *Nature*, **296**, 754.

RÖLLINGHOFF, M., PFIZENMEIER, K., TROSTMANN, H. & WAGNER, H. (1975). T cell proliferation in the mixed lymphocyte culture does not necessarily result in the generation of cytotoxic T effector cells. *Eur. J. Immunol.*, **5**, 560.

SYRJÄNEN, K.J. (1980). Spleen white pulp morphology as an indicator of the immunological state in DBA/2 mice bearing mastocytoma. *Exp. Pathol.*, **18**, 223.